# Designing home-based physical activity programs for rural cancer survivors: A survey of technology access and preferences

**DOI:** 10.3389/fonc.2023.1061641

**Published:** 2023-01-25

**Authors:** Elizabeth A. Salerno, Rohana Gao, Jason Fanning, Neha P. Gothe, Lindsay L. Peterson, Allison B. Anbari, Maura M. Kepper, Jingqin Luo, Aimee S. James, Edward McAuley, Graham A. Colditz

**Affiliations:** ^1^ Division of Public Health Sciences, Department of Surgery, Washington University School of Medicine in St. Louis, St. Louis, MO, United States; ^2^ Alvin J. Siteman Cancer Center, Washington University School of Medicine in St. Louis, St. Louis, MO, United States; ^3^ Academic Program of Medical Education, Washington University School of Medicine in St. Louis, St. Louis, MO, United States; ^4^ Department of Gerontology, Wake Forest School of Medicine, Winston-Salem, NC, United States; ^5^ Department of Kinesiology and Community Health, University of Illinois at Urbana-Champaign, Champaign, IL, United States; ^6^ Beckman Institute for Advanced Science and Technology, University of Illinois at Urbana-Champaign, Urbana, IL, United States; ^7^ Division of Medical Oncology, Department of Medicine, Washington University School of Medicine in St. Louis, St. Louis, MO, United States; ^8^ Sinclair School of Nursing, University of Missouri, Columbia, MO, United States; ^9^ Prevention Research Center, Brown School, Washington University in St. Louis, St. Louis, MO, United States

**Keywords:** physical activity, cancer survivorship, technology, rural, survey, intervention design

## Abstract

**Background:**

While technology advances have increased the popularity of remote interventions in underserved and rural cancer communities, less is understood about technology access and preferences for home-based physical activity programs in this cancer survivor population.

**Purpose:**

To determine access, preferences, and needs, for a home-based physical activity program in rural cancer survivors.

**Methods:**

A Qualtrics Research Panel was recruited to survey adults with cancer across the United States. Participants self-reported demographics, cancer characteristics, technology access and usage, and preferences for a home-based physical activity program. The Godin Leisure Time Exercise Questionnaire (GLTEQ) assessed current levels of physical activity. Descriptive statistics included means and standard deviations for continuous variables, and frequencies for categorical variables. Independent samples t-tests explored differences between rural and non-rural participants.

**Results:**

Participants (N=298; mean age=55.2 ± 16.5) had a history of cancer (mean age at diagnosis=46.5), with the most commonly reported cancer type being breast (25.5%), followed by prostate (16.1%). 74.2% resided in rural hometowns. 95% of participants reported accessing the internet daily. On a scale of 0-100, computer/laptop (M=63.4) and mobile phone (M=54.6) were the most preferred delivery modes for a home-based physical activity intervention, and most participants preferred balance/flexibility (72.2%) and aerobic (53.9%) exercises. Desired intervention elements included a frequency of 2-3 times a week (53.5%) for at least 20 minutes (75.7%). While there were notable rural disparities present (e.g., older age at diagnosis, lower levels of education; *p*s<.001), no differences emerged for technology access or environmental barriers (*p*s>.08). However, bias due to electronic delivery of the survey should not be discounted.

**Conclusion:**

These findings provide insights into the preferred physical activity intervention (e.g., computer delivery, balance/flexibility exercises) in rural cancer survivors, while highlighting the need for personalization. Future efforts should consider these preferences when designing and delivering home-based interventions in this population.

## Introduction

The benefits of physical activity (PA) after a cancer diagnosis (1–4) are significant but rarely realized, as up to two-thirds of survivors remain insufficiently active (5, 6). Traditional PA interventions designed to improve health during survivorship are center-based and effective at managing function, mental health, quality of life, and fatigue (7–9). Emerging evidence has highlighted growing health disparities in rural cancer communities with little to no access to health programs (10, 11); rural survivors tend to be less active than their urban counterparts (12, 13) and are at increased risk of chronic conditions and morbidity (12, 14–16). Unfortunately, the design of traditional PA interventions aimed at eliminating health disparities may reduce access for those cancer survivors who may stand to benefit the most (e.g., rural, functionally impaired/disabled) (10). The lack of accessible and effective home-based physical activity programs (HBPAP) for rural cancer survivors is a critically unmet need (17).

As we consider the design and delivery of such programs, it is important to understand the access barriers specific to rural cancer survivors (18). Technological advances in recent years have expanded the media through which PA programs can be delivered in low-access or low-resource settings (19); however, there is concern that leveraging such technologies may exacerbate existing health disparities (20). The Pew Research Center has highlighted a rapidly closing gap in technology access between rural and non-rural Americans as of 2019 (21), but there are limited data in technology access in rural cancer survivors specifically (22–24).

PA preferences in rural cancer survivors are arguably as important as technology access (25). While survivors generally prefer home-based activities with individual tailoring (22), less is known about the extent to which these preferences also apply to rural cancer survivors. Vallance and colleagues (26) conducted a survey of rural breast cancer survivors in Canada and reported that no PA counseling or programming variables were overwhelmingly endorsed, highlighting that a “one size fits all” approach is likely not suitable for this population (27). Indeed, “rurality” is not a monolith (28), and more nuanced surveys in these populations are warranted.

To address these gaps, we conducted a survey of predominantly rural adult cancer survivors across the United States. This survey aimed to: 1) ascertain key wants and needs for a HBPAP in this population and; 2) identify demographic, behavioral, and preference differences between cancer survivors who live in rural vs. more urban environments. We hypothesized that the majority of cancer survivors would be interested in having access to a HBPAP, with needs for unique cancer-specific features (e.g., exercise modifications) and differences based on residential location.

## Methods

### Participants and study design

To be eligible to participate in the study, individuals must have been panel members in Qualtrics who self-reported a previous cancer diagnosis. Qualtrics is a survey sampling and administration company that was contracted to recruit participants and deploy an internet-based survey; this survey suite is made available to faculty and staff through a university-wide site license. With over 20 online sample providers, Qualtrics recruits participants from traditional, actively managed market research panels and targets recruitment based on contractual need. In the current study, Qualtrics targeted individuals residing in rural locations using a 50% quota for sex. The study team designed the survey online in Qualtrics to be primarily quantitative with a limited number of open-ended questions that allowed for qualitative responses. Survey protocols were reviewed and approved by university Institutional Review Boards (protocol #202102086) and the requirement for consent signature documentation was waived.

### Development of the analytic sample

Responses from 315 individuals with a history of cancer were collected through Qualtrics. To reduce the potential of confounding due to favorable prognosis, we excluded individuals who reported diagnosis of non-melanoma skin cancer or basal cell carcinoma (n=16). Two different methods were used to ensure unique IDs: (1) Qualtrics’ own duplicate-finding system, and; (2) “*percentmatch*” program in Stata (29) to identify the percentage of shared responses between respondents. In potential duplicate pairs with over 95% shared responses, the ID with the least information was excluded (n=1), leaving a final analytic sample of 298 cancer survivors.

## Measures

### Demographic and cancer-specific characteristics

Participants self-reported their current age, gender (male, female, prefer not to say, self-describe), race (American Indian, Asian, Native Hawaiian or Other Pacific Islander, Black, White, More than one race), household income (categories of $15,000 from 0 to ≥$90,000), highest level of education (categories spanning <high school to PhD or equivalent), employment status (part time, full time, retired not working, retired working part time, unemployed, full time homemaker, other), height, weight, and residential location (urban: city or metropolitan; suburban: outskirts of city; rural: outside of towns and cities, sparsely populated). Participants further reported their cancer type(s), age at diagnosis, cancer stage, recurrence (yes/no), current treatment (y/n), and ever receipt of chemotherapy, radiation, or surgery (y/n).

### Technology and environmental access

Participants were asked to answer a series of questions regarding access to technology, including internet, television, streaming services, activity trackers (e.g., Fitbit), mobile phones, computers/laptops, and tablets. Further questions such as frequency of use, ownership vs. sharing, mobile phone type and primary usage were prompted using skip logic depending on previous access responses. Participants were also prompted to report any environmental barriers that may prevent them from being as physically active as they would like, including walkability, access to recreation facilities, safety, transportation, and other.

### Current PA behavior

The Godin Leisure Time Exercise Questionnaire (GLTEQ) (30) was adapted for the Qualtrics survey to assess participants’ current exercise behavior, given our interest in designing exercise-specific programming. Participants indicated the frequency of time spent over the past 7 days engaging in ≥15 minutes of strenuous (e.g., running), moderate (e.g., fast walking), and mild/light exercise (e.g., easy walking). Each activity category was multiplied by nine, five, and three metabolic equivalent of task (MET) hours/week, respectively, and then summed for a total exercise score. Total scores classified individuals as active enough for meaningful health benefits (>24), moderately active (14-24), or insufficiently active (<14) (30).

### PA beliefs and preferences

Participants were asked if they believed cancer patients and survivors “should be physically active” as well as their interest in having access to a HBPAP. Those who were in favor of a HBPAP (n=230) received further questions regarding program preferences, including goals for completion, PA type (e.g., aerobic, balance), intervention tailoring, program frequency, session duration, delivery method (e.g., computer, DVD), and a free-response question for additional comments and preferences.

## Data analysis

Descriptive statistics were calculated for all questionnaire data, including means and standard deviations for continuous variables and frequencies for categorical variables. Missing data was minimal at <5% for any given question, and thus treated as missing at random. Self-reported rurality was dichotomized (yes/no) by collapsing urban and suburban into one category. Reported height and weight outliers that fell outside of biological plausibility were excluded (n=32) prior to the creation of body mass index (BMI) using the standard equation (kg/m^2^) (31) and removed from BMI-specific calculations; however, these participants’ responses to other questions were still included in analyses. T-tests compared differences in continuous demographics, clinical factors, technology access, and PA preferences by rurality. Chi-square tests further compared differences in categorical factors by rurality. All analyses were conducted in SPSS (Version 27; IBM Corp. Armonk, NY), Stata version 17 (StataCorp, College Station, Texas), and R [version 1.4.1717]. Significance level was set to 0.05.

## Results

### Demographic and cancer-specific characteristics

Participant characteristics are detailed below in **Table 1**. Briefly, participants were cancer survivors who were on average 55.2 ± 16.5 years old at survey administration with a majority female (57.4%), white (90.3%) and overweight (BMI *M*=29.0 ± 7.2). 49.7% were college graduates and 40.9% were working at least part-time. 74.2% resided in rural locations, defined as outside towns and cities that are sparsely populated and not within commuting distance (self-reported). The most commonly reported cancer type was breast (25.2%), followed by prostate (16.1%) and cervical (15.1%). Of the 298 cancer survivors, 48 (16.1%) reported multiple cancer types, in which breast, kidney, prostate and lung cancer had the most overlap. Details of cancer types are outlined in **Table 2**. Compared with non-rural survey respondents, participants from rural areas were significantly older at the time of the survey and cancer diagnosis (*p*s<.001). Fewer rural participants graduated college (*p*<.001), made at least $90,000 per year (*p*<.001), worked at least part-time (*p*<.001), and in general reported lower cancer stages (*p*<.001). A smaller percentage of rural participants reported ever receiving chemotherapy or radiation (*p*s<.04) or a cancer recurrence (*p*<.001).

**Table 1:** Participant characteristics.

	Total Sample N=298	Rural n=221	Non-Rural n=77	
	M (SD) or %	M (SD) or %	M (SD) or %	*p* value
Age at survey administration	55.2 (16.5)	58.3 (15.4)	46.5 (16.5)	<0.001
Female	57.4%	57.9%	55.8%	0.72
Race				0.03
White	90.3%	92.8%	83.1%	
Black	3.4%	0.9%	6.5%	
American Indian/Alaskan Native	2.3%	1.4%	2.6%	
Asian	1.7%	2.7%	5.2%	
More than one race	0.7%	0.9%	0%	
Prefer not to answer	1.7%	1.4%	2.6%	
Body mass index ^Ŧ^	29.0 (7.2)	29.43 (6.9)	27.2 (8.1)	0.06
Annual household income ≥$90,000	22.8%	16.3%	41.6%	<.001
College graduate	49.7%	43.4%	67.5%	<.001
Working at least part-time	40.9%	32.6%	64.9%	<.001
Age at diagnosis	46.5 (18.6)	49.0 (17.8)	38.8 (19.2)	<.001
Cancer stage				<.001
I	38.9%	43.9%	24.7%	
II	26.5%	21.7%	40.3%	
III	14.1%	13.1%	16.9%	
IV	3.7%	2.3%	7.8%	
Unknown	16.8%	19.0%	10.4%	
Cancer recurrence	22.5%	13.6%	48.1%	<.001
Ever received chemotherapy	23.8%	23.1%	26.0%	0.04
Ever received radiation	32.6%	33.5%	29.9%	0.03

^Ŧ^ n=32 biologically implausible values excluded.

**Table 2:** Cancer types reported by current sample.

	Total Sample N=298
	n (%)
Breast	75 (25.2%)
Prostate	48 (16.1%)
Cervical	45 (15.1%)
Melanoma	29 (9.7%)
Kidney	26 (8.7%)
Lung	21 (7.0%)
Endometrial/Uterine	16 (5.4%)
Colon or Rectal	20 (6.7%)
Leukemia	18 (6.0%)
Head & Neck	18 (6.0%)
Thyroid	15 (5.0%)
Bladder	14 (4.7%)
Ovarian	10 (3.4%)
Non-Hodgkin’s Lymphoma	8 (2.7%)
Liver	8 (2.7%)
Bone	3 (1.0%)
Other	11 (3.7%)
Multiple	48 (16.1%)

Counts and percentages do not add up to 298 and 100, respectively, due to reported multiple cancers.

### Technology and environmental access

The majority of participants reported owning a television (97.7%) and using the internet daily or at least 5 times a week (95%). Of the 99% who reported access to an electronic device (92.2% mobile phone, 84.1% computer/laptop, 56.9% tablet), most reported owning the device (95.3%) rather than sharing. Of those who owned mobile phones, 86.1% used smartphones with touchscreen and internet access. 64% of mobile phone owners did not use activity tracking apps. Making/receiving calls, sending messages, and surfing the internet were the top reported mobile phone services used in the last 3 months. 35.7% reported an environmental barrier to being physically active; the most commonly reported barrier was walkability (limited access to sidewalks or parks; 20.4%). Rural participants did not differ from their non-rural counterparts on access to technology or environmental barriers (*p*s>.08).

### Current PA behavior

**Figure 1:**
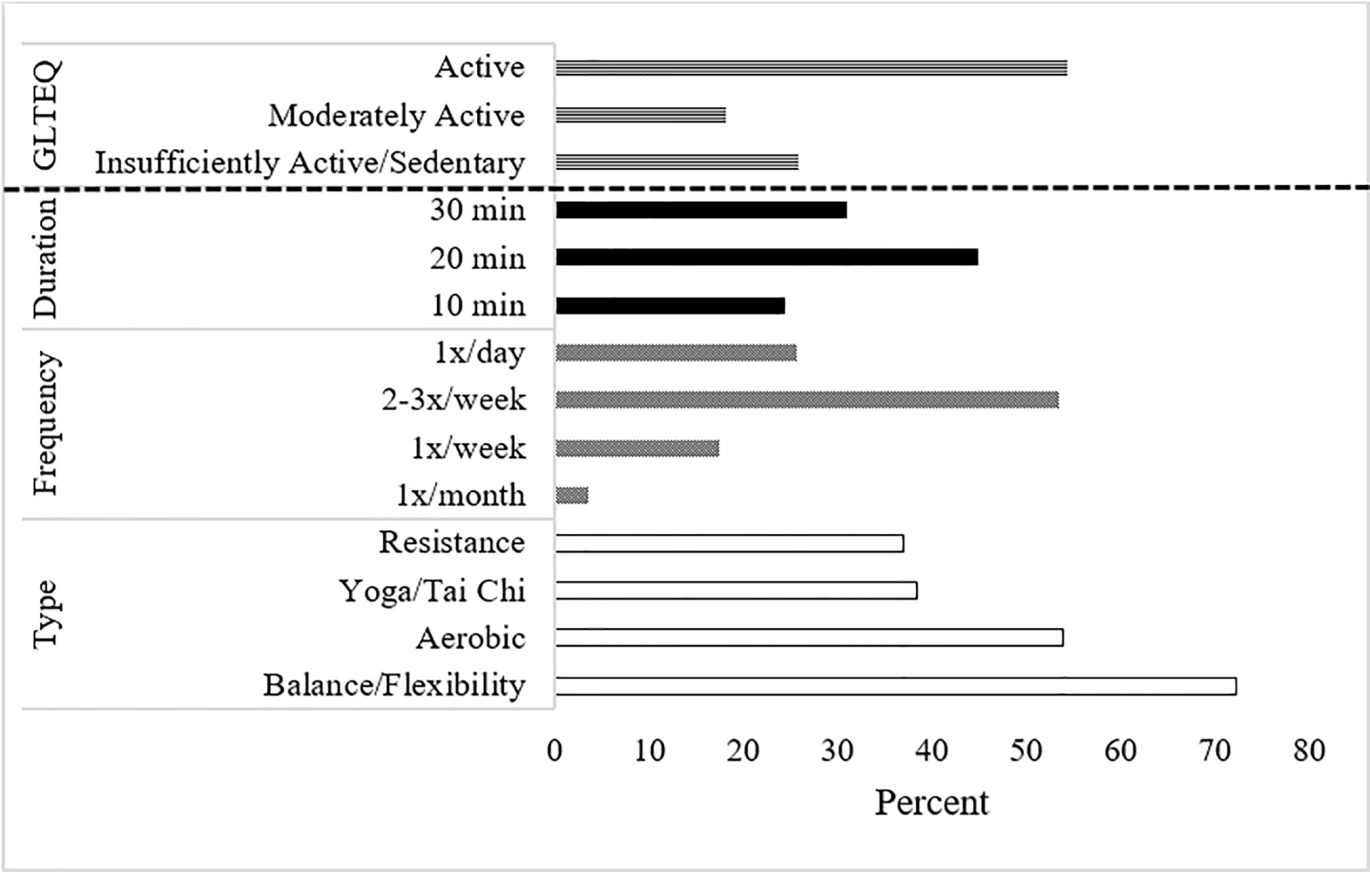
Current physical activity behavior and preferences for a future physical activity program.

Most participants were active (54.4%), 18.1% were moderately active, and 25.0% were insufficiently active/sedentary, as scored by the GLTEQ (see first section of **Figure 1**). When examining each activity intensity category individually, participants reported an average of 1.6, 2.3, and 3 days per week of strenuous, moderate, and light PA, respectively. Rural participants also reported fewer days per week of vigorous (*p*<.001) intensity PA.

### PA interest

Almost all cancer survivors (99%) believed that cancer patients and survivors should be physically active, and 77.2% were interested in a HBPAP. The most commonly cited goals for completing a PA program were: “become healthier overall” (77.4%), “help with fatigue and feel more energized” (63.9%), “feel better about myself” (60%), and “lose weight” (57.4%). 95.6% of participants believed that a PA program would be at least moderately effective for achieving these goals.

### Preferences for home-based PA

Most participants were interested in balance/flexibility (72.2%), followed by aerobic (53.9%), yoga/Tai Chi (38.3%), and resistance/weight lifting (37%) activities (see last three sections of **Figure 1**). Most participants preferred a standard program without cancer-specific tailoring (60.9%), because they “don’t want to think about [their] diagnosis” (45.0%). Of those individuals who preferred a tailored program, they preferred a program that “understands what [they’ve] been through” (84.4%). Half of participants reported willingness to use a HBPAP 2-3 times a week (53.5%) for at least 20 minutes per session (75.7%). On a scale of 0-100, most participants preferred delivery *via* a computer or laptop (M=63.4). 77.0% of participants reported no foreseen difficulties accessing a PA program delivered *via* the above-mentioned media, and 53.0% of participants reported that internet access was not required to increase/optimize use. Most participants were interested in remote delivery of counseling/guidance about how to be active safely (68.7%), with preference for email (M=66.5), text (M=62.9), and Zoom/Skype (M=51.6) delivery. Half were interested in access to a support group of other survivors (50.4%). No rural differences emerged for preferred PA modality (*p*s>.23) or frequency (*p*=.51), although a slightly larger percentage of rural participants preferred shorter PA sessions (*p*=.05). Compared with non-rural participants, rural participants had a lower preference for DVD (*p*=.001) and mobile phone delivery (*p*=.01), and a smaller percentage of rural participants preferred access to a support group and PA tracker to be more active (*p*s<.001).

## Discussion

There is an urgent need to achieve equitable access to PA programs in underserved, rural cancer communities. To design for sustainability and reduce health disparities, it is important to consider these survivors’ technology access and preferences at the outset. Our findings suggest that rural cancer survivors are interested in receiving HBPAPs and that survivors in the current sample have the technological means for successfully participating in such programs. Caution is warranted, however, in placing too much emphasis on these results due to our survey’s digital delivery. These findings also highlight notable differences in demographic and clinical factors between rural and non-rural survivors, underscoring the importance of accelerating our progress towards equitable access to PA programs in these communities.

Over 77% of participants were interested in a HBPAP to become healthier, feel more energized and better in general, and lose weight, all of which are consistent with the exercise oncology literature (1, 22). The most common preferred exercise regimen (e.g., 2-3 days/week of balance exercises and aerobic walking) in this sample of rural cancer survivors is also the same frequency, intensity, and modality that has been demonstrated in the literature to provide important health benefits. A recent roundtable report by the American College of Sports Medicine (ACSM) (1, 2) and updated PA guidelines by the American Cancer Society (ACS) (32) report significant health benefits with moderate-intensity activity up to 3 times per week, including reduced anxiety, depressive symptoms, fatigue, and lymphedema, and improved health-related quality of life and physical function. Observational studies have further reported on survival benefits with increasing levels of post-diagnosis PA (9, 33, 34). Consideration should be given to these preferred features when designing home-based interventions for rural cancer survivors, knowing that they are also likely to provide substantial health benefits.

These preferences for a HBPAP did not differ by rurality, although our results underscore the need for individual tailoring to support survivors’ needs. A smaller percentage of rural participants were interested in a support group, PA trackers, and remote activity counseling compared with their non-rural counterparts. More broadly, half of the current sample preferred a cancer-specific program, while another half did not. Despite 99% believing that cancer survivors should be physically active, only half of participants were classified as “active” by the GLTEQ. This contradiction, accompanied by varied support interests, stresses the importance of identifying PA interventions that are of high value and can be consistently utilized by cancer survivors. One approach may be designing flexible programs (i.e., different programmatic tracks) that prescribe beneficial PA while also meeting participants’ individual needs. These findings support the notion that there is no “one size fits all” approach for rural cancer survivors (26, 35), stressing the need to understand what tools work for whom, in what context, at what time (36). This emphasis on personalization and equity in intervention design draws on principles of behavioral (37) and implementation science (38, 39), signifying a shift away from traditional approaches and toward addressing the unique needs that arise from multiple spheres of influence (40). Researchers should be thoughtful about what ancillary support tools they provide and consider bringing in end users during the design phase to draw on their expertise and systematically assess their needs. This is a key principle of designing for dissemination, implementation, and sustainability (D4DIS) (38) to ensure that we are building effective, sustainable, and equitable programs.

Our finding of widespread access to and use of several technological services and devices is consistent with recent reports on the closing technology gap across populations. Roughly 7 in 10 rural Americans report having a broadband internet connection at home (41), and rural adults have seen almost a 10% increase in stable internet access since 2016 (21). Rural survivors in this sample reported similar internet access as their non-rural counterparts, highlighting this closing technology gap. While the COVID-19 pandemic forced many into technologies they may not have used previously (42), our recruitment processes may also at least partially explain the high levels of reported technology access in this sample. All participants were active panel members for Qualtrics, and thus were more likely to have stable internet access. It is also important to clearly acknowledge that participants in this sample are groups already well represented in research (e.g., White, educated, female), and we must do better in reaching rural cancer survivors who have been historically underserved.

Of further interest are clear differences in several demographic and clinical factors in this sample by rural status. Compared to non-rural participants, rural survivors were older at age of cancer diagnosis and had lower vigorous PA levels. Rural participants also reported lower levels of education and annual household incomes. Fewer rural survivors reported ever receiving chemotherapy or radiation, which could be explained by older age at diagnosis, lower cancer stage reported by rural survivors in this sample, or limited access to the appropriate treatments. There is conflicting evidence that rural cancer survivors present at different stages of disease than their urban counterparts (43–46), which speaks to the diversity of rurality and its clinical implications across the United States. While no rural-specific environmental barriers emerged in this sample, our results underscore the presence of health disparities in rural communities (12, 46, 47) and emphasize the need to improve access to safe and enjoyable PA programs in rural settings.

These results should be interpreted in the context of their strengths and limitations. We surveyed approximately 300 mostly rural cancer survivors to understand technology access and preferences for a HBPAP. This analysis had no cancer type, diagnosis, or treatment restrictions, enrolling a wider variety of survivors than have historically been represented. However, this sample was comprised of predominantly White panel members for Qualtrics, and some important perspectives may not have been captured using this approach. Investigators should replicate and extend these results using different methodologies across varying geographical regions with more questions about clinical factors (e.g., type of chemotherapy) and behaviors (e.g., smoking history). The inclusion of qualitative elements could also explore rich contextual factors that drive technology access and PA preferences and spotlight other determinants that are difficult to measure in surveys, such as systemic racism, stigma, and culture. By design, these data were self-reported and thus may be prone to misclassification. Self-reported measures of physical activity can result in overestimation (48) and are generally designed to capture only one domain of health-enhancing activity; future studies should consider other activity assessment tools (e.g., accelerometry, 24-hour recall) and demographic data (e.g., occupation) to characterize other important domains and contexts of physical activity behaviors in rural cancer communities.

Increasing equitable access to PA programs after a cancer diagnosis should be a top priority, with a special focus on how to leverage technology to reduce health disparities. Our findings provide preliminary feasibility for rural cancer survivors’ interest in, access to, and preferences for a HBPAP that is comprised of balance/flexibility or aerobic walking exercises, at least twice a week for at least 20 minutes a session, and delivered *via* computer or laptop. These preferences are consistent with cancer-specific PA guidelines for the accrual of multiple health benefits and should therefore be given strong consideration, alongside personalization, in the design and development of future HBPAPs in rural cancer survivors.

## Data availability statement

The raw data supporting the conclusions of this article will be made available by the authors, without undue reservation.

## Ethics statement

The studies involving human participants were reviewed and approved by Institutional Review Board at Washington University School of Medicine in St. Louis (protocol #202102086). Written informed consent for participation was not required for this study in accordance with the national legislation and the institutional requirements.

## Author contributions

ES had full access to all the data in the study and takes responsibility for the integrity of the data and the accuracy of the data analysis. Concept and design, ES, EM, and GC. Acquisition, analysis, and interpretation of data, ES and RG. Drafting of the manuscript, ES and RG. Critical revision of the manuscript for important intellectual content, all authors. Statistical analysis, ES and RG. Obtained funding, N/A. Administrative, technical, or material support, GC. Supervision, EM and GC. All authors contributed to the article and approved the submitted version.

## References

[1] Campbell KL Winters-Stone KM Wiskemann J May AM Schwartz AL Courneya KA. Exercise guidelines for cancer survivors: Consensus statement from international multidisciplinary roundtable. Med Sci Sports Exerc (2019) 51(11):2375–90. doi: 10.1249/MSS.0000000000002116 PMC8576825 31626055

[2] Patel AV Friedenreich CM Moore SC Hayes SC Silver JK Campbell KL. American College of sports medicine roundtable report on physical activity, sedentary behavior, and cancer prevention and control. Med Sci Sports Exerc (2019) 51(11):2391–402. doi: 10.1249/MSS.0000000000002117 PMC6814265 31626056

[3] Speck RM Courneya KS Mâsse LC Duval S Schmitz KH. An update of controlled physical activity trials in cancer survivors: A systematic review and meta-analysis. J Cancer Surviv (2010) 4(2):87–100. doi: 10.1007/s11764-009-0110-5 20052559

[4] Kushi LH Doyle C McCullough M Rock CL Demark-Wahnefried W Bandera EV. American Cancer society guidelines on nutrition and physical activity for cancer prevention: Reducing the risk of cancer with healthy food choices and physical activity. CA Cancer J Clin (2012) 62(1):30–67. doi: 10.3322/caac.20140 22237782

[5] Tarasenko Y Chen C Schoenberg N. Self-reported physical activity levels of older cancer survivors: Results from the 2014 national health interview survey. J Am Geriatr Soc (2017) 65(2):e39–44. doi: 10.1111/jgs.14589 27943255

[6] Mason C Alfano CM Smith AW Wang C-Y Neuhouser ML Duggan C. Long-term physical activity trends in breast cancer survivors. Cancer Epidemiol Biomarkers Prev (2013) 22(6):1153–61. doi: 10.1158/1055-9965.EPI-13-0141 PMC3688258 23576689

[7] Schmitz KH Campbell AM Stuiver MM Pinto BM Schwartz AL Morris GS. Exercise is medicine in oncology: Engaging clinicians to help patients move through cancer. CA Cancer J Clin (2019) 69(6):468–84. doi: 10.3322/caac.21579 PMC7896280 31617590

[8] Alfano CM Bluethmann SM Tesauro G Perna F Agurs-Collins T Elena JW. NCI funding trends and priorities in physical activity and energy balance research among cancer survivors. JNCI J Natl Cancer Inst (2016) 108(1):285. doi: 10.1093/jnci/djv285 26547926

[9] Mctiernan A Friedenreich CM Katzmarzyk PT Powell KE Macko R Buchner D. Physical activity in cancer prevention and survival: A systematic review. Med Sci Sports Exerc (2019) 51(6):1252–61. doi: 10.1249/MSS.0000000000001937 PMC6527123 31095082

[10] Charlton M Schlichting J Chioreso C Ward M Vikas P. Challenges of rural cancer care in the united states (2015). Available at: https://www.cancernetwork.com/oncology-journal/challenges-rural-cancer-care-united-stateshttps://www.cancernetwork.com/oncology-journal/challenges-rural-cancer-care-united-states2/7 (Accessed August 11, 2021). 26384798

[11] Williams F Jeanetta S James AS. Geographical location and stage of breast cancer diagnosis: A systematic review of the literature. J Health Care Poor Underserved (2016) 27(3):27. doi: 10.1353/hpu.2016.0102 27524773

[12] Weaver KE Palmer N Lu L Case LD Geiger AM. Rural-urban differences in health behaviors and implications for health status among US cancer survivors. Cancer Causes Control (2013) 24(8):1481–90. doi: 10.1007/s10552-013-0225-x PMC3730816 23677333

[13] Zahnd WE James AS Jenkins WD Izadi SR Fogleman AJ Steward DE. Rural–urban differences in cancer incidence and trends in the united states. Cancer Epidemiol Prev Biomarkers (2018) 27(11):1265–74. doi: 10.1158/1055-9965.EPI-17-0430 PMC5787045 28751476

[14] Burris JL Andrykowski M. Disparities in mental health between rural and nonrural cancer survivors: A preliminary study. Psychooncology (2010) 19(6):637–45. doi: 10.1002/pon.1600 PMC2880195 19582800

[15] Yabroff KR Han X Zhao J Nogueira L Jemal A. Rural cancer disparities in the united states: A multilevel framework to improve access to care and patient outcomes. JCO Oncol Pract (2020) 16(7):409–13. doi: 10.1200/op.20.00352 32574130

[16] Singh GK Siahpush M. Widening rural–urban disparities in all-cause mortality and mortality from major causes of death in the USA, 1969–2009. J Urban Heal (2014) 91(2):272–92. doi: 10.1007/s11524-013-9847-2 PMC3978153 24366854

[17] Mama SK Lopez-Olivo MA Bhuiyan N Leach HJ. Effectiveness of physical activity interventions among rural cancer survivors: A systematic review and meta-analysis. Cancer Epidemiol Biomarkers Prev (2021) 30(12):2143–53. doi: 10.1158/1055-9965.EPI-21-0871/671152/AM/EFFECTIVENESS-OF-PHYSICAL-ACTIVITY-INTERVENTIONS PMC8643319 34620628

[18] Morris BB Rossi B Fuemmeler B. The role of digital health technology in rural cancer care delivery: A systematic review. J Rural Heal (2022) 38(3):493–511. doi: 10.1111/JRH.12619 PMC8894502 34480506

[19] Arigo D Jake-Schoffman DE Wolin K Beckjord E Hekler EB Pagoto SL. The history and future of digital health in the field of behavioral medicine. J Behav Med (2019) 42(1):67–83. doi: 10.1007/S10865-018-9966-Z 30825090 PMC6644720

[20] Arcaya MC Figueroa JF. Emerging trends could exacerbate health inequities in the united states. Health Affairs (2017) 36(6):992–8. doi: 10.1377/HLTHAFF.2017.0011 28583956

[21] Some digital divides between rural, urban, suburban America persist . Pew Research Center. Available at: https://www.pewresearch.org/fact-tank/2021/08/19/some-digital-divides-persist-between-rural-urban-and-suburban-america/ (Accessed September 15, 2021).

[22] Wong JN McAuley E Trinh L. Physical activity programming and counseling preferences among cancer survivors: A systematic review. Int J Behav Nutr Phys Act (2018) 15(1):1–21. doi: 10.1186/s12966-018-0680-6 29879993 PMC5992647

[23] Phillips SM Conroy DE Keadle SK Pellegrini CA Lloyd GR Penedo FJ. Breast cancer survivors’ preferences for technology-supported exercise interventions. Support Care Cancer (2017) 25(10):3243–52. doi: 10.1007/s00520-017-3735-3 PMC5832636 28470368

[24] Phillips SM Courneya KS Welch WA Gavin KL Cottrell A Nielsen A. Breast cancer survivors’ preferences for mHealth physical activity interventions: Findings from a mixed methods study. J Cancer Surviv (2019) 13(2):292–305. doi: 10.1007/s11764-019-00751-3 30912011 PMC6499383

[25] Lewis BA Williams DM Frayeh A Marcus BH. Self-efficacy versus perceived enjoyment as predictors of physical activity behaviour. Psychol Heal (2016) 31(4):456–69. doi: 10.1080/08870446.2015.1111372 PMC4769927 26541890

[26] Vallance JK Lavallee C Culos-Reed NS Trudeau MG. Predictors of physical activity among rural and small town breast cancer survivors: An application of the theory of planned behaviour. Psychol Heal Med (2012) 17(6):685–97. doi: 10.1080/13548506.2012.659745 22409699

[27] Ginossar T Brakey HR Sussman AL Price B Kano M Davis S. “You’re going to have to think a little bit different” barriers and facilitators to using mHealth to increase physical activity among older, rural cancer survivors. Int J Environ Res Public Heal (2021) 18(17):8929. doi: 10.3390/IJERPH18178929 PMC8430471 34501517

[28] Leider JP Meit M McCullough JM Resnick B Dekker D Alfonso YN. The state of rural public health: Enduring needs in a new decade. Am J Public Health (2020) 110(9):1283–90. doi: 10.2105/AJPH.2020.305728 PMC7427223 32673103

[29] Kuriakose N Robbins M. Don’t get duped: Fraud through duplication in public opinion surveys 1. Statistical Journal of the IAOS (2016) 32(3):283–291. doi: 10.3233/SJI-160978

[30] Shephard R. Godin leisure-time exercise questionnaire. Med Sci Sport Exerc (1997) 29(6):S36–8.

[31] World Health Organization. Physical status: The use and interpretation of anthropometry (1995). World Heal Organ - Tech Rep Ser. Available at: https://apps.who.int/iris/bitstream/handle/10665/37003/W?sequence=1 (Accessed August 2, 2022). 8594834

[32] Rock CL Doyle C Demark-Wahnefried W Meyerhardt J Courneya KS Schwartz AL. Nutrition and physical activity guidelines for cancer survivors. CA Cancer J Clin (2012) 62(4):243–74. doi: 10.3322/caac.21142 22539238

[33] Irwin ML Smith AW McTiernan A Ballard-Barbash R Cronin K Gilliland FD. Influence of pre- and postdiagnosis physical activity on mortality in breast cancer survivors: the health, eating, activity, and lifestyle study. J Clin Oncol (2008) 26(24):3958–64. doi: 10.1200/JCO.2007.15.9822 PMC2654316 18711185

[34] Cao C Friedenreich CM Yang L. Association of daily sitting time and leisure-time physical activity with survival among US cancer survivors. JAMA Oncol (2022) 8(3):395–403. doi: 10.1001/jamaoncol.2021.6590 PMC8739832 34989765

[35] Nielsen AM Welch WA Gavin KL Cottrell AM Solk P Torreg EA. Preferences for mHealth physical activity interventions during chemotherapy for breast cancer: A qualitative evaluation. Support Care Cancer (2020) 28(4):1919–28. doi: 10.1007/s00520-019-05002-w PMC6992480 31367917

[36] Norman GJ. Answering the “What works?” question in health behavior change. Am J Prev Med (2008) 34(5):449–50. doi: 10.1016/J.AMEPRE.2008.02.005 PMC2405809 18407014

[37] Bandura A. Social cognitive theory: An agentic perspective. Annu Rev Psychol (2001) 52(1):1–26. doi: 10.1146/annurev.psych.52.1.1 11148297

[38] Kwan BM Brownson RC Glasgow RE Morrato EH Luke DA. Designing for dissemination and sustainability to promote equitable impacts on health. Annual Review of Public Health (2022) 43(1):331–53. doi: 10.1146/ANNUREV-PUBLHEALTH-052220-112457 PMC9260852 34982585

[39] Brownson RC Jacobs JA Tabak RG Hoehner CM Stamatakis KA. Designing for dissemination among public health researchers: Findings from a national survey in the united states. American Journal of Public Health (2013) 103(9):1693–9. doi: 10.2105/AJPH.2012.301165 PMC3966680 23865659

[40] Peterson A Charles V Yeung D Coyle K. The health equity framework: A science- and justice-based model for public health researchers and practitioners. Health Promot Pract (2021) 22(6):741–6. doi: 10.1177/1524839920950730 PMC8564233 32814445

[41] Demographics of Internet and home broadband usage in the united states . Pew Research Center. Available at: https://www.pewresearch.org/internet/fact-sheet/internet-broadband/ (Accessed September 15, 2021).

[42] The Internet and the pandemic . Pew Research Center. Available at: https://www.pewresearch.org/internet/2021/09/01/the-internet-and-the-pandemic/ (Accessed September 22, 2022).

[43] Shugarman LR Sorbero MES Tian H Jain AK Ashwood JS. An exploration of urban and rural differences in lung cancer survival among medicare beneficiaries. Am J Public Health (2008) 98(7):1280–7. doi: 10.2105/AJPH.2006.099416 PMC2424098 17971555

[44] Monroe AC Ricketts TC Savitz LA. Cancer in rural versus urban populations: A review. J Rural Heal (1992) 8(3):212–20. doi: 10.1111/J.1748-0361.1992.TB00354.X 10121550

[45] Paquette I Finlayson SRG. Rural versus urban colorectal and lung cancer patients: Differences in stage at presentation. J Am Coll Surg (2007) 205(5):636–41. doi: 10.1016/J.JAMCOLLSURG.2007.04.043 17964438

[46] Nguyen-Pham S Leung J McLaughlin D. Disparities in breast cancer stage at diagnosis in urban and rural adult women: A systematic review and meta-analysis. Ann Epidemiol (2014) 24(3):228–35. doi: 10.1016/j.annepidem.2013.12.002 24462273

[47] Meilleur A Subramanian SV Plascak JJ Fisher JL Paskett ED Lamont EB. Rural residence and cancer outcomes in the united states: Issues and challenges. Cancer Epidemiol Biomarkers Prev (2013) 22(10):1657–67. doi: 10.1158/1055-9965.EPI-13-0404 PMC3814162 24097195

[48] Dyrstad SM Hansen BH Holme IM Anderssen SA. Comparison of self-reported versus accelerometer-measured physical activity. Med Sci Sport Exerc (2014) 46(1):99–106. doi: 10.1249/MSS.0b013e3182a0595f 23793232

